# Association between short-term exposure to ambient air pollutants and the risk of hospital visits for acute upper respiratory tract infections among adults: a time-series study in Ningbo, China

**DOI:** 10.1186/s12889-024-19030-7

**Published:** 2024-06-10

**Authors:** Jin-Ying Huang, Wei Feng, Guo-Xin Sang, Stuart McDonald, Tian-Feng He, Yi Lin

**Affiliations:** 1 Nottingham Ningbo GRADE Centre, School of Economics, Faculty of Humanities and Social Sciences, University of Nottingham, Ningbo, China; 2 Fenghua District Center for Disease Control and Prevention, Ningbo, China; 3 Ningbo Municipal Center for Disease Control and Prevention, 1166, Fanjiangan Road, Ningbo, 315010 China; 4 Department of Occupational and Environmental Health Sciences, School of Public Health, Peking University, Beijing, China

**Keywords:** Acute upper respiratory tract infection, Hospital visits, Ambient air pollutant, Time-series analysis, China

## Abstract

**Objectives:**

Acute upper respiratory tract infections (AURTIs) are prevalent in the general population. However, studies on the association of short-term exposure to air pollution with the risk of hospital visits for AURTIs in adults are limited. This study aimed to explore the short-term exposure to air pollutants among Chinese adults living in Ningbo.

**Methods:**

Quasi-Poisson time serious regressions with distributed lag non-linear models (DLNM) were applied to explore the association between ambient air pollution and AURTIs cases. Patients ≥ 18 years who visit three hospitals, being representative for urban, urban–rural junction and rural were included in this retrospective study.

**Results:**

In total, 104,441 cases with AURTIs were enrolled in hospital during 2015–2019. The main results showed that particulate matter with an aerodynamic diameter less than 2.5 μm (PM_2.5_), nitrogen dioxide (NO_2_) and nitrogen dioxide (SO_2_), were positively associated to hospital visits for AURTIs, except for nitrogen dioxide (O_3_), which was not statistically significant. The largest single-lag effect for PM_2.5_ at lag 8 days (RR = 1.02, 95%CI: 1.08–1.40), for NO_2_ at lag 13 days (RR = 1.03, 95%CI: 1.00–1.06) and for SO_2_ at lag 5 days (RR = 1.27, 95%CI: 1.08–1.48), respectively. In the stratified analysis, females, and young adults (18–60 years) were more vulnerable to PM_2.5_ and SO_2_ and the effect was greater in rural areas and urban–rural junction.

**Conclusions:**

Exposure to ambient air pollution was significantly associated with hospital visits for AURTIs. This study provides epidemiological evidence for policymakers to control better air quality and establish an enhanced system of air pollution alerts.

**Supplementary Information:**

The online version contains supplementary material available at 10.1186/s12889-024-19030-7.

## Introduction

Acute respiratory infections (ARIs) are a major global public health concern, leading to high morbidity [1, 2]. Based on the site of infection, acute upper respiratory tract infections (AURTIs) originate from the airways that extend from the nose to the voice cords within the larynx [3]. Typical symptoms of AURTIs include coughing, sore throat, nasal obstruction, and headache. AURTIs are prevalent in the general population, with the number of reported cases exceeding 17 billion in 2019 [4–7]. There was a huge burden of AURTIs cases in China, which accounted for 15% of the global incidence of AURTIs in 2019 [8]. Although the mortality rate due to AURTIs is relatively small, it has an adverse effect on daily life and constitutes a significant burden on health care system [9, 10].

With the rapid economic growth, urbanization, industrialization and transportation development in the past four decades, air pollution has become an increasing serious environmental problem in China [4]. A large number of studies have linked respiratory infection with exposure to ambient air pollutants [10–13]. Previous Chinese studies conducted in Beijing, Shanghai, Wuhan, and Lanzhou have shown positive associations between the exposure to ambient air pollutants and hospital visits for ARIs [4, 11–13]. For instance, a 10 ug/m^3^ increase in concentration of PM_10_, S0_2_, and NO_2_ were associated with 1.72%, 1.34% and 2.57% increases respectively in respiratory disease emergency admissions in Beijing. However, difference in the source of air pollutants, environmental factors, population density, pathogen identification across various location may affect duration and severe levels of ARIs.

Most of the studies have primarily focused on the association between air pollution and respiratory illnesses. However, research on the negative impacts of air pollution on AURTIs is notably scarce in Western countries and even more so in China. In addition, the incidence of respiratory infection caused by air pollutants varies in demographic information [14, 15]. Most prior research have focused on the impacts of children and the elderly and paid less attention to the adults [7, 15–17]. To the best of the authors’ knowledge, only two observational studies have been conducted in Ningbo; these two studies have focused exclusively on children [18] and the adults aged 65 and over [19]. However, the exposure pattern is different among children and the elderly from working age adults. Few studies are updated and available on the association between outdoor air pollution and AURTIs in adults in Zhejiang province. Thus, the object of the study was to investigate the effect of short-term exposure ambient air pollutants on hospital visits for AURTIs among Chinese adults in Ningbo, stratified by sex, age and geographical centers located in Fenghua district.

## Methods

### Study area

Ningbo is one of the important industrial cities in the coastal area of the Yangtze River Delta. Having the world’s highest cargo throughput port, the city features heavy industry such as manufacturing and chemical processing and refining [20]. This study was conducted in Fenghua district, located in the southern region of Ningbo. As of 2022, Fenghua district had a population of 478,000 residents [21]. Ningbo experiences a mild and humid subtropical monsoon climate, featuring short springs and autumns, prolonged winters and summers, abundant sunshine, and ample rainfall.

### Hospitalization data and patient enrollment

**Fig. 1:**
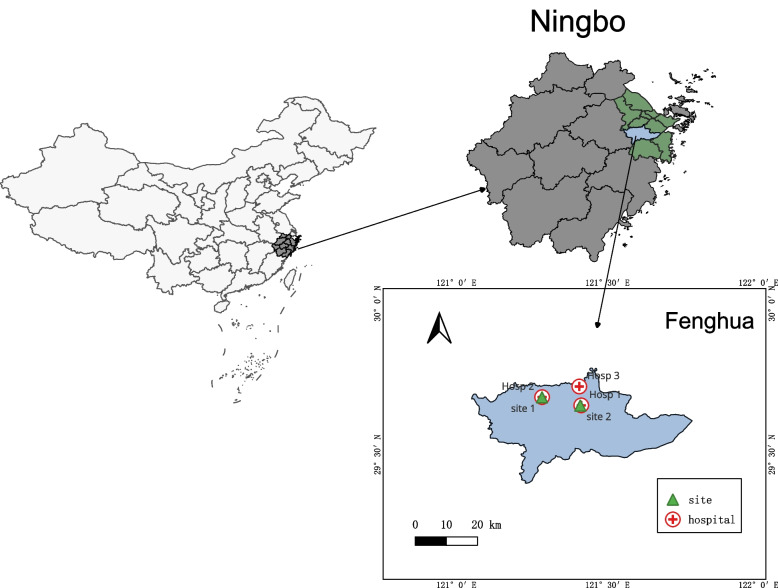
The geographical location of monitoring sites and sampling hospitals in Fenghua district, Ningbo

Jiangkou, Song’ao and Shangtian hospitals were selected to be representative for local hospitals in urban, urban–rural junction, and rural areas (Fig. 1). The data on the number of patients visiting hospital for AURTIs and relevant information were obtained from January 2015 to December 2019. AURTIs was screened and coded using the international classification of diseases (ICD 10) codes J00-J99: diseases of the respiratory diseases [22]. Specifically, the hospital visits of adults for AURTIs data were classified into AURTIs (J00-J06). AURTIs consist of acute nasopharyngitis (J00), acute sinusitis (J01), acute pharyngitis (J02), acute tonsillitis (J03), acute laryngitis and tracheitis (J04), acute obstructive laryngitis and epiglottitis (J05) and acute upper respiratory infections of multiple and unspecified sites (J06).

Adults visiting hospitals for the first time to examine AURTIs were included in this study providing they met the following criteria: (1) patients had symptoms of acute infection (e.g., hypothermia); (2) patients had symptoms of having sore throat, runny nose, cough and shortness of breath; (3) patients had symptoms of acute asthma; (4) patients completed demographic and medical history questionnaires. The exclusion criteria for selected target patients were: (1) patients aged under 18 years; (2) patients who was not local residents in Fenghua district; (3) patients diagnosed with chronic respiratory diseases; (4) patients having any acute respiratory cancers; (5) patients re-visiting for health examination of respiratory infections.

### Air pollution and meteorological data

Data on air pollution and meteorological data were obtained from the Environment Monitoring Center of Ningbo. Two monitoring sites, Fenghua Xikou government and Fenghua Environmental Protection Bureau, were selected to collect air pollution in urban and suburban areas of this district (Fig. 1). This study focused on four ambient air pollutants: PM_2.5_, NO_2_, SO_2_, and O_3_. The daily concentrations of these air pollutants were calculated by average data from each of the monitoring sites. Meteorological data included daily mean temperature (MT, °C), daily mean relative humidity (RH, %) and daily mean wind speed (WS, m/s).

### Statistically analysis

The associations between air pollutants and health outcomes are often characterized by lag effects. This means that potentially complex temporal patterns of risk arising from time-varying exposures need to be taken into consideration [23]. A commonly used method to investigate these temporal patterns is the distributed lag non-linear model (DLNM), which is used to conduct time series analysis. Due to hospital admission for AURTIs are generally considered exist over-dispersed, the generalized linear model (GLM) based on Quasi-Poisson distribution is employed to fit the non-linear mixed factors [24]. Specifically, we adopt a natural cubic splines (*ns*) function to control the long-term trends, seasonal variation, meteorological factors, impact of day of the week, and holiday effect. That is, “*time*” for long-term trends and seasonal variation; MT, RH, and WS for meteorological factors; “*day of week (DOW)*” for the impact of the day of the week. “*holiday*” for whether it is holiday or not.

$$Yt\mathit\sim quasi\mathit\;Poission\;\left(\mathrm{\mu t}\right)$$

$$\text{log}\left({\upmu\mathrm{t}}\right)=\beta+W_X^T\eta+ns\left(time,7/years\right)+ns\left(MT,{df}_1\right)+ns\left(RH,{df}_2\right)+ns\left(WS,{df}_3\right)+as.factors\left(DOW\right)+as.factors(holidays)$$

Where $Yt$ is the actual cases of AURTI in $t$ days, $\mathrm{\upmu{t}}$ is the expected cases of AURTI in 𝑡 days. ${W}_{X}^{T}\eta$ and $ns$ represent the cross-basis function and natural cubic function respectively, $df_1$ , $df_2$ and $df_3$ denote the degree of freedom in terms of meteorological factors, $\beta$ is the intercept.

According to modified Akaike information criteria (AIC) for models with over-dispersed responses fitted through quasi-likelihood, we have based the choice of the number of splines, which defines the df in each dimension, given by:

$$QAIC=-2 \mathcal{L}\left(\widehat{\theta }\right)+2\widehat{\phi }k$$

Where $\mathcal{L}$ is the log-likelihood of the fitted model with parameters $\widehat{\theta }$ and $\widehat{\phi }$ the estimated overdispersion parameter, whereas $k$ is the number of parameters. The best model is chosen that minimizes the criteria above [23]. Table A1 shows the Q-AIC value for the different lag and df for time among air pollutants. The value of Q-AIC for optimal lag days is ranging from 14 to 27 days. We also select the two—pollutant model (NO_2_ and SO_2_) by using Q-AIC in Table A2.

Relative risk (RR) was used to capture the short-term effect of exposure to air pollution, which means that the lag-specific and cumulative risk of AURTIs hospital cases per 10 ug/m^3^ increase in air pollutants concentrations.

A single-lag model was constructed to explore the potential delayed influence of air pollution on outpatient visits. Given that the health effects of air pollution could last for several days, cumulative-lag effects were included to evaluate the cumulative effects of air pollution. The reference levels to evaluate the effects of air pollutants exposure on AURTIs patient visits is based on class II levels of National Environmental Quality Standards (GB3095-2012). That is, levels reference given at 35 μg/m^3^ for PM_2.5_, 40 μg/m^3^ for NO_2_, 50 μg/m^3^ for SO_2_, 160 μg/m^3^ for O_3_, respectively.

Sensitivity analyses were performed to examine the robustness of our findings. Two-pollutant model was used to estimate the association between combination of two pollutants and hospital visits for AURTIs. Second, different degree of freedom (4-5df) in the ns function for the meteorological factors were examined.

All analyses were performed with DLNM package in R software version 2.4.7. Significance level $\alpha =0.05$, except where otherwise reported.

## Results

### Descriptive results

The total number of AURTIs cases from 2015 to 2019 was 104,441 (male: 51.89%), with a mean of 57 daily visits (11–174) (Table 1). When stratified by age and geographical centers, 34.08% of total cases were over 60 years, and 62.41% were living in rural areas, respectively.

**Table 1:** Summary statistics of air pollutants, meteorological factors, and AURTIs, 2015–2019

	NO. (%)	Mean	SD	Centiles				
				Min	25%	75%	Max	IQR
**AURTIs**	104,441	57.39	26.89	11	39	68	174	29
**Sex**								
Male	54,192 (51.89)	29.78	14.12	3	20	37	94	17
Female	50,249 (48.11)	27.61	13.91	3	18	34	97	16
**Age groups**								
18–60	68,850 (65.92)	37.83	18.83	6	24	47	122	23
≥ 60	35,591 (34.08)	19.56	10.58	1	12	24	75	12
**Center**								
Urban area	19,652 (18.82)	10.80	8.83	0	4	15	60	9
Urban–rural junction	19,610 (18.77)	10.78	6.15	0	6	14	42	8
Rural area	65,179 (62.41)	35.81	18.59	3	23	43	120	20
**Air pollutants**								
PM_2.5_		35.29	23.98	4	19	44	224	25
NO_2_		37.12	17.24	5	24	48	118	24
SO_2_		10.16	5.34	3	7	12	60	5
O_3_		63.45	28.79	3	43	83	164	40
**Meteorological factors**								
Mean temperature (MT, °C)		17.91	8.22	-4.3	10.8	24.4	33.0	13.7
Relative humidity (RH, %)		73.93	12.10	28	66	83	97	17
Wind speed (WS, m/s)		10.28	6.87	0	6	12	60	6

*AURTIs* Acute upper respiratory tract infections, *SD* Standard Deviation, *IQR* Interquartile Range, *PM*_*2.5*_ Particulate matter with an aerodynamic diameter less than 2.5 μm, *NO*_*2*_ Nitrogen dioxide, *SO*_*2*_ Sulfur dioxide, *O*_*3*_ Ozone, *MT* Mean temperature, *RH* Relative humidity, *WS* Wind speed

Regarding the meteorological indicators, the daily mean concentration of PM_2.5_ was 35.29 μg/m^3^ (from 4 to 244 μg/m^3^), 37.12 μg/m^3^ for NO_2_ (from 5 to 118 μg/m^3^), 10.16 μg/m3 for SO_2_ (from 3 to 60 μg/m^3^), and 63.45 μg/m^3^ for O_3_ (from 3 to 164 μg/m^3^), respectively. In addition, the average daily concentration of MT, RH, and WS was 17.91℃, 73.93%, and 10.28 m/s, respectively.

**Fig. 2:**
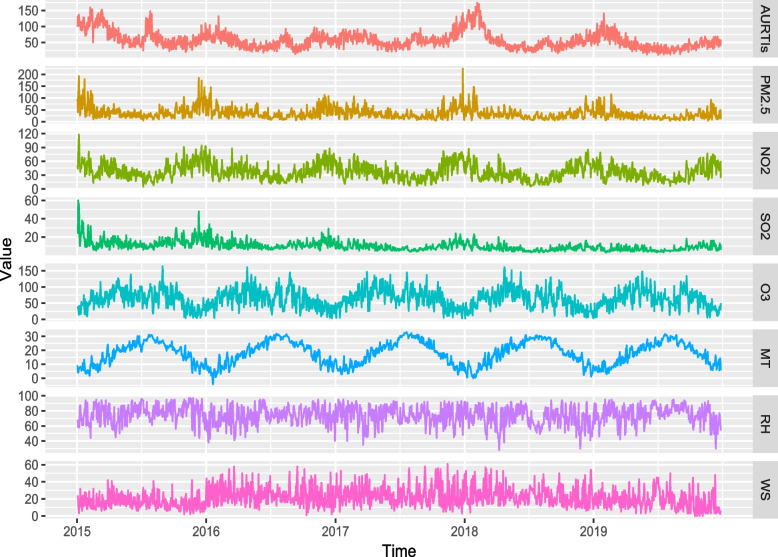
Daily distribution of AURTIs, meteorological factors, and air pollutants from 2015 to 2019. Abbreviations as in Table 1

Figure 2 presents the daily air pollutants, meteorological factors, and AURTIs hospital visits from 2015 to 2019. It can be observed that the daily average concentrations of air pollutants in Ningbo were higher in spring, and winter compared to summer and autumn.

### Correlation between air pollutants and meteorological factors

Spearman rank correlation between meteorological factors and air pollutants is presented in Table A3. PM_2.5_, NO_2_, and SO_2_ showed positive correlations with each other, with the exception of O3. Additionally, Spearman's correlation coefficients between these meteorological factors and air pollutants are less than 0.75, indicating that current covariate selection could avoid possible multicollinearity Spearman’s correlation.

### The effects of air pollutants on the risk of hospital visits for AURTIs

**Fig. 3:**
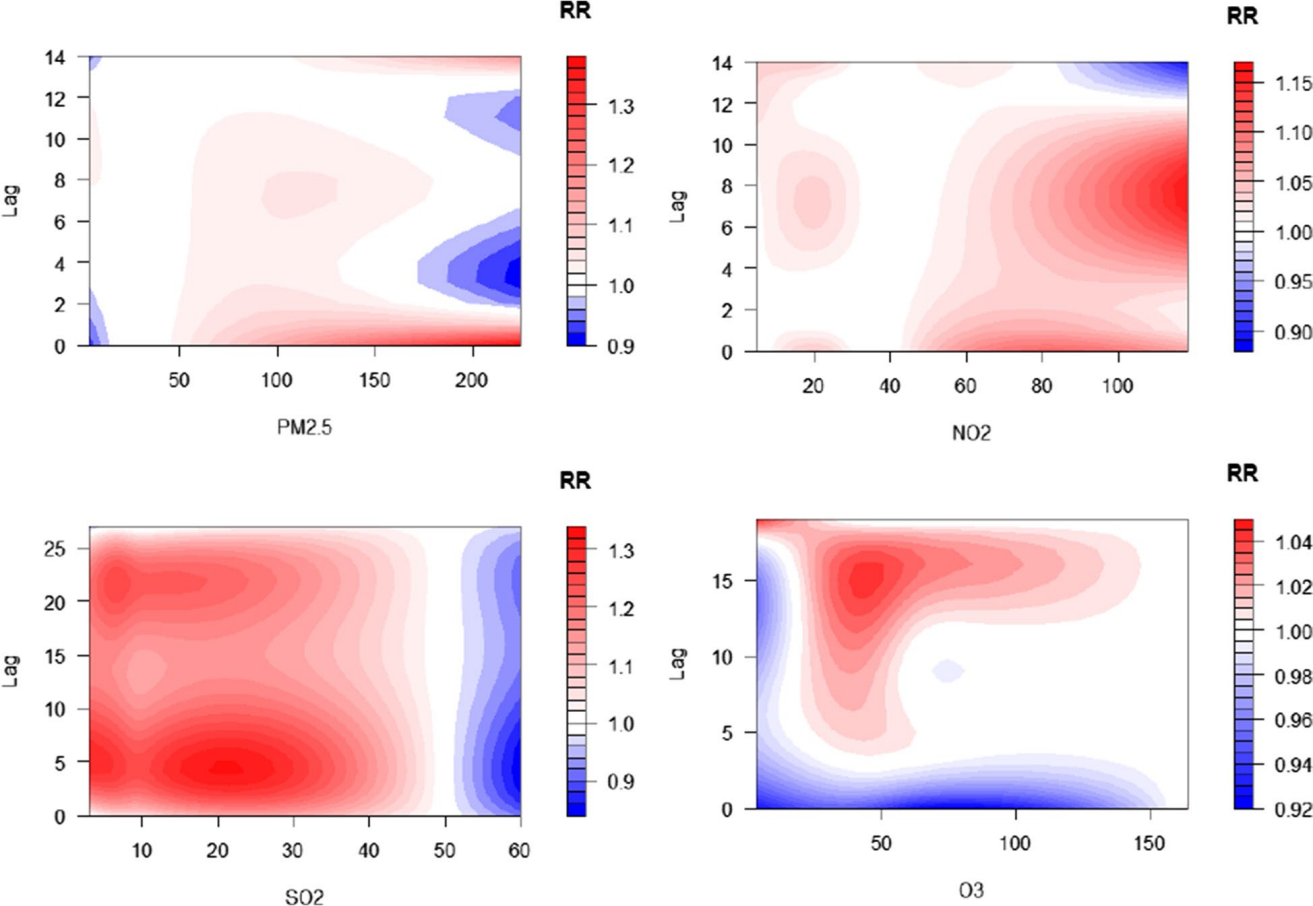
Contour plots for relative risks of AURTIs hospital visits along ambient air pollutant at lag periods. Abbreviations as in Table 1

Figure 3 presents a comprehensive summary of the bi-dimensional exposure-lag-response relationships between air pollution exposure and risk of hospital visits for AURTIs across varying lag days. Overall, exposure to PM_2.5_, NO_2_ and SO_2_ was associated with increased risk for hospital visits for AURTIs. However, O_3_ exposure was not significantly associated with risk of hospital visits for AURTIs.

**Fig. 4:**
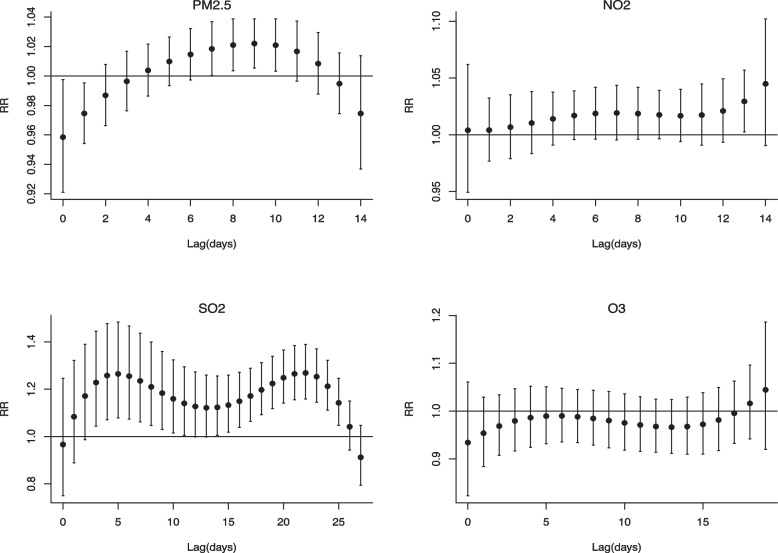
Lag-specific relative risks in outpatient visits for AURTIs per 10-unit increase in daily mean concentrations of air pollutants on the single pollutant model. Abbreviations as in Table 1. Reference at 35 μg/m^3^ for PM_2.5_, 40 μg/m^3^ for NO_2_, 50 μg/m^3^ for SO_2_, 160 μg/m^3^ for O_3_, respectively

Figure 4 illustrates the single-lag effect of four ambient air pollutants on the relative risk of hospital visits for AURITs. PM_2.5_ had a significant effect in increasing the risk of hospital visits for AURTI (i.e., relative risk, RR > 1), from lag 7 days (RR = 1.018, 95%CI: 1.001–1.037) to lag 10 days (RR = 1.021, 95%CI: 1.003–1.039). Similarly, a 10-unit increase in NO_2_ concentration was associated with an increased risk of hospital visits for AURTIs. The single-lag effect of NO_2_ was significant at lag 13 days (RR = 1.029, 95%CI: 1.003–1.057). The risk for the effect of SO_2_ increased at longer lags, ranging from lag 3 days (RR = 1.228, 95%CI: 1.044–1.445) to lag 25 days (RR = 1.041, 95%CI: 1.047–1.245). However, the estimates for the effect of O_3_ effect were found to be insignificant.

Table 2 summaries the cumulative-lag effects of four ambient air pollutants. The cumulative-lag effect of PM_2.5_ (lags 0–14 days) was found to be statistically insignificant. Conversely, the cumulative-lag effect of NO_2_ was significant at lags 0–14 days (RR = 1.293, 95%CI: 1.069–1.562). SO_2_ had the maximum cumulative-lag effect at lags 0–27 days (RR = 5.713, 95%CI: 1.041–31.361). No significant cumulative-lag effect was observed for O_3_.

**Table 2:** Relative risk with 95% CI of daily hospital visits for AURTIs stratified by sex, age, and center associated with 10-unit increase of air pollutant from single-pollutant model

	PM_2.5_^a^		NO_2_^b^		SO_2_^c^		O_3_^d^	
	RR	CI	RR	CI	RR	CI	RR	CI
All	1.02	(0.87, 1.19)	1.29	(1.07, 1.56)^*^	4.81	(1.10, 21.04)^*^	0.67	(0.31, 1.44)
Male	1.02	(0.86, 1.21)	1.18	(0.96, 1.46)	2.83	(0.51, 15.79)	0.65	(0.28, 1.51)
Female	1.02	(0.85, 1.22)	1.42	(1.14, 1.76)^*^	7.53	(1.52, 37.22)^*^	0.70	(0.29, 1.65)
18–60	1.05	(0.88, 1.24)	1.26	(1.02, 1.54)^*^	5.88	(1.25, 27.68)^*^	0.66	(0.29, 1.51)
≥ 60	0.97	(0.80, 1.17)	1.37	(1.09, 1.72)^*^	2.21	(0.30, 16.57)	0.71	(0.28, 1.83)
Urban	0.94	(0.65, 1.35)	1.17	(0.74, 1.82)	2.20	(0.09, 54.07)	0.96	(0.83, 1.12)
Urban–rural junction	1.10	(0.87, 1.39)	1.37	(1.02, 1.83)^*^	5.48	(0.55, 54.90)	0.39	(0.12, 1.27)
Rural	1.01	(0.85, 1.20)	1.30	(1.05, 1.60)^*^	5.71	(1.04, 31.36)^*^	0.68	(0.29, 1.60)

*RR* Relative risk, *CI* Confidence level

^*^P < 0.05

^a^The model shows the maximum lag of 14 days for PM_2.5_

^b^The model shows the maximum lag of 14 days for NO_2_

^c^The model shows the maximum lag of 27 days for SO_2_

^d^The model shows the maximum lag of 19 days for O_3_

### The effects of air pollutants on the risk of AURTIs hospital visits by sex, age, and geographical center

**Fig. 5:**
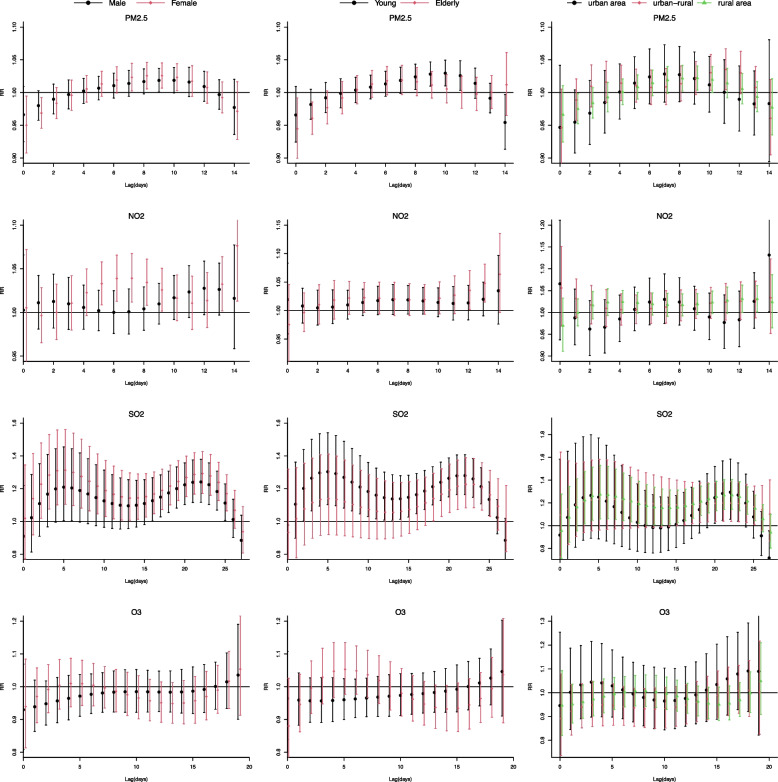
Lag-specific relative risks in hospital visits for AURTIs per 10 unit increase in single-pollutant models stratified by sex, age and geographic center. Abbreviations as in Table 1

After the stratified analysis, Fig. 5 shows the single-lag effect on hospital visits for AURTIs per a 10-unit increase in single-pollutant models, stratified by sex, age, and geographical center. The harmful effects of PM_2.5_ and NO_2_ were stronger in females, indicating that females were more vulnerable to ambient air pollution than males. In terms of SO_2_, both females and males were significantly associated with ambient air pollution, with effects beginning at lag 3 days in females and lag 17 days in males. For age subgroup, only 18–60 years remained significantly associated with PM_2.5_, whereas over 60 years groups was statistically significant with NO_2_ and SO_2_, with effects beginning at lag 12 days and 19 days, respectively. O_3_ was not found to be associated with any age groups. In terms of geographical center, PM_2.5_ exposure increased risk of AURTIs for hospital visits in rural areas from lag 8 days (RR = 1.021, 95%CI 1.002–1.041) to lag 10 days (RR = 1.020, 95%CI 1.000–1.040). Furthermore, residents at the urban–rural junctions also experienced a delayed effect from lag 10 to 12 days. Residents in rural areas were at higher risk of hospital visits for AURTIs due to exposure to SO_2_, with the largest RR at lag 5 days (RR = 1.273, 95%CI 1.060–1.523).

The cumulative-lag effects for the stratified analysis are shown in Table 2. In the age-stratified analysis, only for NO_2_ was found to be associated with hospital visits for AURITs in both groups of 18–60 years and over 60 years at lags 0–14 days, with RR 1.255 (95%CI 1.019–1.544) and RR 1.370 (95%CI 1.088–1.723), respectively. When stratified by geographic center, the cumulative-lag effect of NO_2_ exposure remained statistically significant in both urban–rural junctions and rural area. Meanwhile, the associations remained significant only for rural areas (RR = 5.713, 95%CI 1.041–31.361) on the day of exposure to SO_2_. None of the subgroups (sex, age, and geographical center) were found a significant cumulative-lag effect for PM_2.5_ and O_3_. We also report the lag 0-maxium effect (with five days interval for PM_2.5_, NO_2_ and O_3_ and ten days interval for SO_2_) in Table A4.

### Sensitivity analysis

Table 3 provides relative risk with 95% CI of daily hospital visits for AURTIs from two -pollutant model. After adjusting with other air pollutant, the NO_2_ and SO_2_ in the two-pollutant model remained consistent results compared to the single-pollutant model. Figure A1 presents the estimated RR of the lag-specific AURTIs after adjusting df for meteorological factors. The results indicate that using alternative df for mean temperature, relatively humidity, and wind speed did not substantially change the associations between air pollutant levels and hospital visits for AURTIs.

**Table 3:** Relative risk with 95% CI of daily hospital visits for AURTIs from two -pollutant model

	Two-pollutant model	RR	CI	Cumulative RR	CI
PM_2.5_^a^	+ NO_2_	0.94	(0.89,0.98)^*^	0.91	(0.75,1.10)
	+ SO_2_	0.96	(0.92,1.00)	0.97	(0.80,1.15)
	+ O_3_	0.96	(0.92,1.00)	0.97	(0.82,1.14)
NO_2_^b^	+ PM_2.5_	1.09	(1.02,1.16)	1.48	(1.16,1.86)^*^
	+ SO_2_	1.04	(0.98,1.09)	1.42	(1.16,1.73)^*^
	+ O_3_	1.04	(0.98,1.09)	1.26	(1.03,1.52)^*^
SO_2_^c^	+ PM_2.5_	1.06	(1.00,1.10)^*^	5.63	(1.25,25.1)^*^
	+ NO_2_	1.07	(1.01,1.12)^*^	3.86	(0.89,16.7)
	+ O_3_	1.06	(1.01,1.11)^*^	4.71	(1.06,20.8)^*^
O_3_^d^	+ SO_2_	1.05	(0.92,1.19)	0.66	(0.31,1.40)
	+ NO_2_	1.06	(0.93,1.20)	0.45	(0.20,0.97)
	+ O_3_	1.00	(0.88,1.13)	0.43	(0.19,0.92)

*RR* Relative risk; CI: confidence level

^*^P < 0.05

^a^The model shows the maximum lag of 14 days for PM_2.5_

^b^The model shows the maximum lag of 14 days for NO_2_

^c^The model shows the maximum lag of 27 days for SO_2_

^d^The model shows the maximum lag of 19 days for O_3_

## Discussion

This study is the first longitudinal study investigating on the association between short-term exposure to air pollutants and the risk of hospital visits for AURTIs in adults in Zhejiang province, China. Our findings suggest that short-term exposure to ambient air pollutants (PM_2.5_, NO_2_, and SO_2_) were associated with an increased risk of hospital visits for AURTIs among specific demographic groups (sex, age, and geographical center-specific) after controlling for temperature, relative humidity, wind speed, public holiday, and vacation.

Evidence suggested that AURTIs have been identified as a significant threat to the quality of life, human survival, and the burden of diseases on society and government [25–27]. In our study, the total cases of AURTIs among adults were 104,441, with an average of 57 daily hospital visits (range: 11–174). To date, the evidence on AURTIs among adults in the general population remains limited. In a retrospective time-series study in Hong Kong, the mean daily consultations due to AURTIs in general outpatient clinics ranged from 68.4 to 253 from 2008 to 2010 [28]. The lower number of hospital visits for AURTIs in our study might be attributed to the difference in the population demographics.

Many studies have investigated the association between air pollution and lower respiratory diseases [25, 29]. Our study found stronger associations are consistent with previous epidemiological studies in China [18, 27, 30], Europe [31, 32], and USA [33, 34]. PM_2.5_ contains tiny liquid or solid droplets that can be inhaled deeply into human lung and cause serious health effects [35]. We observed a toxic and delayed effect induced by PM_2.5_ starting from lag 7 days. For every 10-unit increase in PM_2.5_ concentrations, the RR of hospital visits for AURTIs increased by 1.018 (95%CI: 1.001–1.037). The harmful effect of particular matter with AURTIs were consistent with previous Chinese studies conducted in Shenzhen [36]. However, one epidemiological study conducted in Jinan suggested that PM_2.5_ had an instant effect [37]. The differences may be attributed to Jinan’s higher latitude, less humid, and colder climate compared to the geography and climate of Ningbo.

Regarding the gaseous pollutants (NO_2_ and SO_2_), we found that exposure to NO_2_ had a significant effect on hospital visits for AURTIs at lag 13 days (RR = 1.029, 95%CI: 1.003–1.057). In contrast to NO_2_, exposure to SO_2_ had an instant effect on hospital visits for AURTIs, lasting from 3–26 days and peaking at lag 22 days (RR = 1.268 95%CI: 1.158–1.389). SO_2_ was the dominant air pollutant causing more hospital visits for AURTIs in our study. Similarly, with limited available studies on AURTIs in China, a study conducted in Wuhan found that SO_2_ had the most significant effect on respiratory disease mortality. For each 10-unit increase in SO_2_, there was a 1.9% increase in RR of overall respiratory disease mortality [38]. However, another Chinese study reported that NO_2_ had the greatest adverse effect than other air pollutants on asthma hospitalization in Shanghai [12]. Such inconsistencies might be attributed to the differences in industrial structure between Shanghai and Ningbo/Wuhan. The primary industries in Ningbo and Shanghai are focusing on manufactures (e.g. automobile manufacture) and financial technology, respectively. Furthermore, the cumulative-lag effect showed a stronger association for gaseous pollutant than particular matter. The maximum cumulative effects of NO_2_ and SO_2_ were significant with RR of 1.293 (95% CI: 1.069–1.562) and 4.809 (95%CI: 1.099–21.041), respectively. In comparison with the findings from single pollutant model, our study also examined the effects of co-pollutants using a two-pollutant model, demonstrating consistent results.

Previous studies investigating the association between short-term O_3_ exposure and hospital visits for AURTIs yielded inconclusive results. O_3_ showed an insignificant associated with AURTIs in our study, consistent with the findings of previous studies conducted in Taiwan and Chongqing [15, 39]. Conversely, a study conducted in Beijing found a negative association between exposure to O_3_ and hospital visits for AURTIs [40]. The potential reason might be that O_3_ can react with other air pollutants to produce new chemical compounds as O_3_’s instability in the Earth’s atmosphere [41].

In the stratified analysis, our results demonstrated that females were more vulnerable to PM_2.5_, NO_2_ and SO_2_. Some studies reported the opposite results that the greatest effects of PM_10_, PM_2.5_ and SO_2_ on boys than girls [14, 15]. One possible explanation is that boys have relatively smaller airways in proportion to their lung volume than girls, while this difference is not observed in adults. Another possible explanation for this phenomenon is that women tend to seek healthcare more often than men [42].

In term of age-stratified analysis, a stronger association between PM_2.5_ and SO_2_ and hospital visits for AURTIs was observed in young adults than the elderly. Young adults have higher exposure to ambient air pollutants, whereas the elderly are most likely to spend 80–90% of their daily time indoor [43]. The higher frequency antibiotic intake among the elderly can prevent AURTIs caused by viruses and bacteria, potentially explaining the fewer hospitals visits in the elderly [44]. Furthermore, the higher rate of free influenza vaccination injection among the elderly is another essential factor, as the free vaccination campaign was promoted to the elderly by the Ningbo government [45, 46].

Furthermore, significant associations between ambient PM_2.5_ and SO_2_ and hospital visits for AURTIs were found in urban–rural junction and rural area. These findings align with a study on the urban–rural disparity in PM_2.5_ and the risk of death from COPD in Chongqing [47]. This can be attributed to several factors. Firstly, the concentrations and compositions air pollutants between urban and rural areas were different [48]. According to the city plan in Ningbo, the majority of manufacturing and industrial activities are located in urban–rural junction areas. Secondly, variations in education levels and health literacy in rural areas may lead to diverse attitudes among local residents towards air pollution control and self-protection practices [16, 49].

The study has several strengths. It represents the first longitudinal study to investigate the association between short-term effect of ambient air pollutants and hospital visits for AURTIs in adults in Zhejiang province. In addition, the study conducted stratified analyses based on large-scale sex, age, and center-specific data for hospital visits. Furthermore, it employed advanced DLNM to consider the lag effect on infection induced by exposure to air pollutants. However, several limitations should be acknowledged. Firstly, we averaged monitoring data for the exposures in Ningbo and did not consider individual exposure to air pollutants, which might affect the accuracy of the main findings. Secondly, some socioeconomic status-related factors and relevant aspects such as the highest education level, occupation, disease history, and medical records were not obtained in our study. Furthermore, since data on PM_2.5_ components is not available, future studies will investigate the association between PM_2.5_ constituents and the risk of hospital visits for AURTIs, aiming to identify the most impactful component. Last but not least is that sources of infection (e.g., cold, bacteria, viruses) were not available, thus, we cannot conduct a stratified analysis by infection source. Future longitudinal studies need to be carried out to examine the association between short-term effect of ambient air pollutants and AURTIs stratified by infection sources.

## Conclusion

Our study provides evidence that increased exposure to PM_2.5_, NO_2_, and SO_2_ contributes to a higher risk of hospital visits for AURTIs. PM_2.5_ showed single-lag effects while NO_2_ and SO_2_ showed both single-lag and cumulative-lag effects. Besides, SO_2_ was the main driver air pollutant driving hospital visits for AURTIs in Ningbo. According to the stratified analysis, females and patients who aged under 60 years and lived in uran-rural junction or rural areas were found to be associated with higher frequency of hospital visits for AURTIs.

### Supplementary Information

Supplementary Material 1.

## Data Availability

The data that support the findings of this study are not openly available due to reasons of sensitivity and are available from the corresponding author upon reasonable request.
