# Supplementary material for: Association between short-term exposure to ambient air pollutants and the risk of hospital visits for acute upper respiratory tract infections among adults: a time-series study in Ningbo, China
Source: BMC Public Health. 2024 Jun 10;24:1555. doi: 10.1186/s12889-024-19030-7 (PMC11163729; doi:10.1186/s12889-024-19030-7)

**Association between short-term exposure to ambient air pollutants and the risk of hospital visits for acute upper respiratory tract infections among adults: A time-series study in Ningbo, China**

Jin-Ying Huang^1^, Wei Feng^2^, Guo-Xin Sang^3^, Stuart McDonald^4^,

Tian-Feng He^3,5*^, Yi Lin^6*^

^1^Nottingham Ningbo GRADE Centre, School of Economics, Faculty of Humanities and Social Sciences, University of Nottingham, Ningbo, China,

^2^Fenghua District Center for Disease Control and Prevention, Ningbo, China

^3^Ningbo Municipal Center for Disease Control and Prevention, Ningbo, China

^4^School of Economics, Faculty of Humanities and Social Sciences, University of Nottingham, Ningbo, China,

^5^Department of Occupational and Environmental Health Sciences, School of Public Health, Peking University, Beijing, China

^6^Centre for Health Economics, School of Economics, Faculty of Humanities and Social Sciences, University of Nottingham, Ningbo, China

*Corresponding Author:

Yi Lin, Center for Health Economics, School of Economics, Faculty of Humanities and Social Sciences, University of Nottingham, Ningbo China. 199, Taikang East Road, University Park, 315100 Ningbo, China

Tel: 0086-574 8818 0009 (8846). E-mail: [Lily.Lin@nottingham.edu.cn](mailto:Lily.Lin@nottingham.edu.cn)

Tian-Feng He, Ningbo Municipal Center for Disease Control and Prevention, Ningbo, China. 1166, Fanjiangan Road, 315010 Ningbo, China

Tel: 0086-13615889754. E-mail: [hetfnbcdc@163.com](mailto:hetfnbcdc@163.com)

**Context**

Table A1 Q-AIC value for the different lag and df for time among air pollutants

Table A2 Q-AIC value for the different df for time among two-pollutants model

Table A3 Spearman rank correlation between meteorological factor and air pollutants

Table A4 Cumulative Relative risk with 95% CI of daily hospital visits for AURTIs stratified by sex, age, and center associated with 10-unit increase of air pollutant from single-pollutant model

Figure A1 The single-pollutant model varying df 3-5 for the meteorological factors.

**Table A1 Q-AIC value for the different lag and df for time among air pollutants**

|  | PM_2.5_ | | | NO_2_ | | | SO_2_ | | | O_3_ | | |
| --- | --- | --- | --- | --- | --- | --- | --- | --- | --- | --- | --- | --- |
| lag | df=7 | df=8 | df=9 | df=7 | df=8 | df=9 | df=7 | df=8 | df=9 | df=7 | df=8 | df=9 |
| 1 | 20496.96 | 20422.31 | 20553.58 | 20621.54 | 20514.69 | 20664.00 | 20685.87 | 20607.26 | 20745.53 | 20847.80 | 20790.08 | 20919.80 |
| 2 | 20451.46 | 20369.30 | 20499.61 | 20603.02 | 20490.22 | 20647.74 | 20625.53 | 20540.76 | 20680.49 | 20846.26 | 20785.83 | 20918.44 |
| 3 | 20407.78 | 20322.78 | 20455.36 | 20538.59 | 20416.57 | 20588.42 | 20564.19 | 20475.73 | 20621.87 | 20842.28 | 20783.76 | 20915.74 |
| 4 | 20377.70 | 20299.36 | 20439.29 | 20518.03 | 20393.13 | 20574.02 | 20517.22 | 20428.67 | 20579.04 | 20835.56 | 20776.61 | 20907.28 |
| 5 | 20359.00 | 20275.88 | 20414.36 | 20495.71 | 20365.01 | 20550.00 | 20449.54 | 20356.06 | 20504.53 | 20805.08 | 20746.62 | 20883.68 |
| 6 | 20351.96 | 20266.80 | 20406.99 | 20465.57 | 20352.34 | 20527.93 | 20415.13 | 20323.57 | 20476.60 | 20756.63 | 20706.23 | 20841.10 |
| 7 | 20312.59 | 20221.95 | 20365.50 | 20414.06 | 20316.21 | 20490.78 | 20311.34 | 20228.58 | 20389.03 | 20759.46 | 20711.21 | 20852.81 |
| 8 | 20278.17 | 20189.66 | 20339.21 | 20392.15 | 20296.76 | 20479.77 | 20246.30 | 20178.17 | 20341.03 | 20716.27 | 20670.05 | 20820.87 |
| 9 | 20268.00 | 20181.79 | 20334.93 | 20351.24 | 20271.94 | 20452.18 | 20208.26 | 20149.92 | 20315.20 | 20702.94 | 20655.65 | 20804.03 |
| 10 | 20233.07 | 20154.71 | 20311.38 | 20323.93 | 20257.15 | 20434.42 | 20166.41 | 20116.21 | 20282.68 | 20687.54 | 20644.88 | 20789.97 |
| 11 | 20250.57 | 20168.56 | 20324.98 | 20315.51 | 20247.74 | 20422.55 | 20145.35 | 20101.84 | 20264.91 | 20662.40 | 20625.58 | 20764.75 |
| 12 | 20237.75 | 20161.97 | 20320.82 | 20325.55 | 20258.45 | 20435.48 | 20089.75 | 20056.79 | 20217.87 | 20646.21 | 20612.04 | 20746.43 |
| 13 | 20219.00 | 20154.25 | 20319.10 | 20286.28 | 20230.76 | 20395.96 | 20061.72 | 20034.74 | 20187.57 | 20649.79 | 20610.19 | 20740.86 |
| 14 | 20220.89 | **20154.16** | 20318.66 | 20272.15 | **20210.96** | 20371.01 | 20047.92 | 20024.02 | 20176.88 | 20565.79 | 20518.36 | 20640.04 |
| 15 | 20225.80 | 20155.34 | 20317.74 | 20293.70 | 20228.45 | 20386.20 | 20022.53 | 20008.12 | 20154.73 | 20578.06 | 20522.56 | 20630.92 |
| 16 |  |  |  |  |  |  | 19965.15 | 19949.75 | 20094.74 | 20541.91 | 20477.18 | 20575.10 |
| 17 |  |  |  |  |  |  | 19939.46 | 19921.10 | 20059.20 | 20541.43 | 20478.24 | 20578.68 |
| 18 |  |  |  |  |  |  | 19949.39 | 19923.80 | 20058.72 | 20530.17 | 20473.67 | 20574.57 |
| 19 |  |  |  |  |  |  | 19941.38 | 19907.56 | 20039.66 | 20498.99 | **20454.96** | 20558.58 |
| 20 |  |  |  |  |  |  | 19911.23 | 19873.67 | 20002.05 | 20506.48 | 20464.28 | 20571.25 |
| 21 |  |  |  |  |  |  | 19855.22 | 19811.82 | 19939.35 |  |  |  |
| 22 |  |  |  |  |  |  | 19788.38 | 19749.23 | 19874.84 |  |  |  |
| 23 |  |  |  |  |  |  | 19788.55 | 19747.52 | 19871.62 |  |  |  |
| 24 |  |  |  |  |  |  | 19749.23 | 19711.59 | 19823.88 |  |  |  |
| 25 |  |  |  |  |  |  | 19755.76 | 19717.16 | 19830.02 |  |  |  |
| 26 |  |  |  |  |  |  | 19716.73 | 19690.23 | 19806.66 |  |  |  |
| 27 |  |  |  |  |  |  | 19671.12 | **19659.74** | 19778.00 |  |  |  |
| 28 |  |  |  |  |  |  | 19680.18 | 19677.56 | 19793.15 |  |  |  |
| 29 |  |  |  |  |  |  | 19734.48 | 19728.72 | 19835.30 |  |  |  |
| 30 |  |  |  |  |  |  | 19737.07 | 19731.03 | 19835.12 |  |  |  |

Note: Q-AIC: Quasi-Akaike’s information criterion; Bold is the lowest value and selected df for the corresponding air-pollutants model.

**Table A2 Q-AIC value for the different df for time among two-pollutants model**

|  | df | | |
| --- | --- | --- | --- |
|  | 7 | 8 | 9 |
| PM_2.5_ + NO_2_ | 20342.16 | 20245.44 | 20264.21 |
| PM_2.5_ + SO_2_ | 19960.21 | 19850.6 | 19812.47 |
| PM_2.5_ + O_3_ | 20348.96 | 20205.4 | 20208.9 |
| NO_2_ + SO_2_ | 19895.4 | 19811.95 | **19807.93** |
| NO_2_ + O_3_ | 20348.96 | 20205.4 | 20208.9 |
| SO_2_ + O_3_ | 19989.57 | 19906.33 | 19897.84 |

Note: Q-AIC: Quasi-Akaike’s information criterion; Bold is the lowest value and selected df for the corresponding two-pollutants model.

|  | PM_2.5_ | NO_2_ | SO_2_ | O_3_ | MT | RH | WS |
| --- | --- | --- | --- | --- | --- | --- | --- |
| PM_2.5_ | 1.0000 |  |  |  |  |  |  |
| NO_2_ | 0.7307^*^ | 1.0000 |  |  |  |  |  |
| SO_2_ | 0.7180^*^ | 0.6727^*^ | 1.0000 |  |  |  |  |
| O_3_ | -0.1118^*^ | -0.4047^*^ | -0.0692^*^ | 1.0000 |  |  |  |
| MT | -0.4677^*^ | -0.5583^*^ | -0.3693^*^ | 0.3618^*^ | 1.0000 |  |  |
| RH | -0.0858^*^ | -0.0384 | -0.0692^*^ | -0.0620^*^ | 0.1023^*^ | 1.0000 |  |
| WS | -0.1418^*^ | -0.1688^*^ | -0.2768^*^ | 0.0557^*^ | 0.0598^*^ | -0.0440^*^ | 1.0000 |

**Table A3 Spearman rank correlation between meteorological factors and air pollutants**

*P<0.05

**Table A4 Cumulative Relative risk with 95% CI of daily hospital visits for AURTIs stratified by sex, age, and center associated with 10-unit increase of air pollutant from single-pollutant model**

|  | Lag | Male | | Female | | 18-60 | | ≥60 | | Urban | | | Urban-rural junction | | Rural | |
| --- | --- | --- | --- | --- | --- | --- | --- | --- | --- | --- | --- | --- | --- | --- | --- | --- |
|  |  | RR | CI | RR | CI | RR | CI | RR | CI | RR | | CI | RR | CI | RR | CI |
| PM_2.5_^a^ | 0-5 | 0.94 | (0.85,1.03) | 0.92 | (0.83,1.01) | 0.95 | (0.86,1.04) | 0.89 | (0.80,0.99) | 0.93 | (0.84,1.02) | | 0.88 | (0.71,1.07) | 0.98 | (0.86,1.12) |
|  | 0-10 | 1.02 | (0.89,1.16) | 1.03 | (0.89,1.18) | 1.06 | (0.93,1.21) | 0.96 | (0.82,1.11) | 1.02 | (0.89,1.17) | | 0.98 | (0.73,1.30) | 1.07 | (0.88,1.28) |
| NO_2_^b^ | 0-5 | 1.04 | (0.92,1.17) | 1.07 | (0.94,1.21) | 1.06 | (0.94,1.20) | 1.05 | (0.91,1.19) | 1.06 | (0.93,1.19) | | 0.97 | (0.74,1.26) | 1.13 | (0.95,1.34) |
|  | 0-10 | 1.08 | (0.91,1.27) | 1.25 | (1.04,1.48)^*^ | 1.16 | (0.97,1.37) | 1.16 | (0.95,1.39) | 1.16 | (0.97,1.37) | | 1.05 | (0.72,1.50) | 1.22 | (0.96,1.55) |
| SO_2_^c^ | 0-10 | 3.76 | (0.57,18.5) | 9.38 | (1.62,14.1)^*^ | 8.07 | (1.46,12.4)^*^ | 2.40 | (0.27,17.1) | 1.56 | (0.57,4.20) | | 1.38 | (0.20,9.13) | 1.74 | (0.44,6.77) |
|  | 0-20 | 13.67 | (0.79,23.5) | 14.49 | (3.53,25.3)^*^ | 11.71 | (3.15,25.2)^*^ | 6.23 | (0.22,20.9) | 3.37 | (0.73,15.4) | | 1.69 | (0.09,29.6) | 3.62 | (0.45,28.6) |
| O_3_^d^ | 0-5 | 0.74 | (0.51,1.07) | 0.93 | (0.62,1.36) | 0.78 | (0.53,1.12) | 0.94 | (0.61,1.43) | 0.82 | (0.56,1.20) | | 1.09 | (0.50,2.35) | 0.66 | (0.39,1.10) |
|  | 0-10 | 0.68 | (0.39,1.17) | 0.86 | (0.48,1.51) | 0.66 | (0.38,1.13) | 1.02 | (0.54,1.90) | 0.83 | (0.47,1.46) | | 1.01 | (0.33,3.03) | 0.47 | (0.21,1.00) |

RR: relative risk; CI: confidence level.

*P<0.05

^a^ The model shows the maximum lag of 14 days for PM_2.5_.

^b^ The model shows the maximum lag of 14 days for NO_2_.

^c^ The model shows the maximum lag of 27 days for SO_2_.

^d^ The model shows the maximum lag of 19 days for O_3_

**Figure A1 The single-pollutant model varying df 3-5 for the meteorological factors**

**NO_2_**


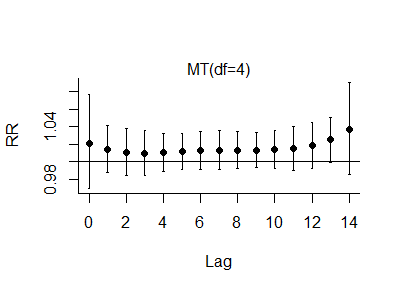

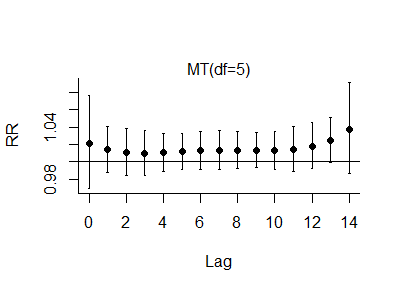

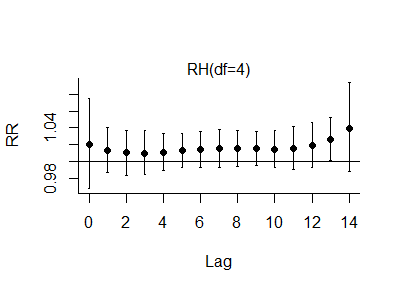

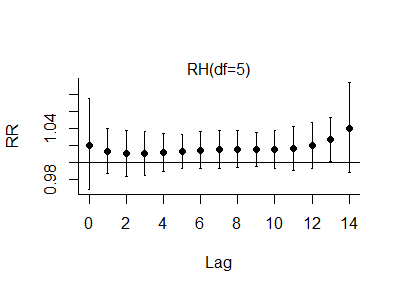

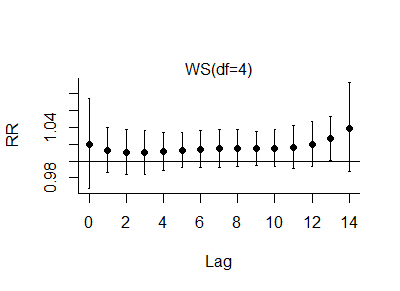

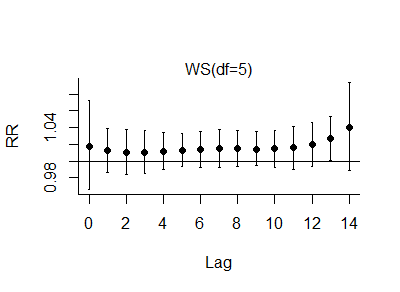


**SO_2_**


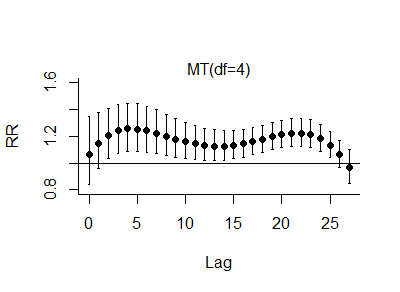

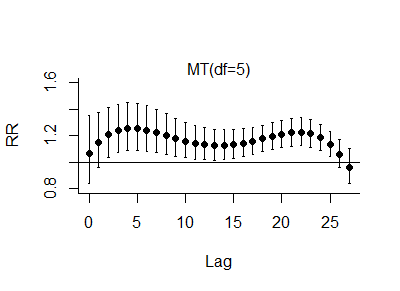

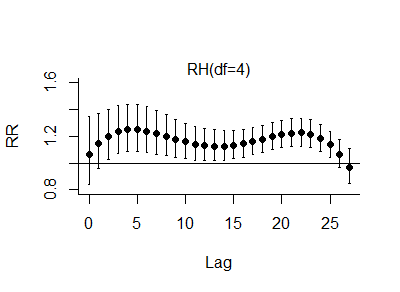

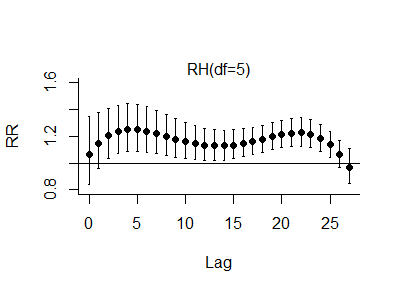

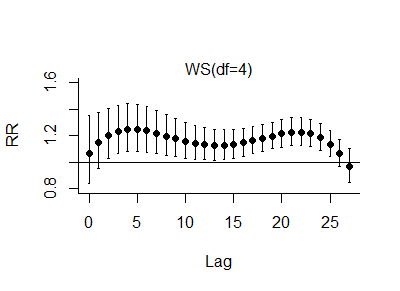

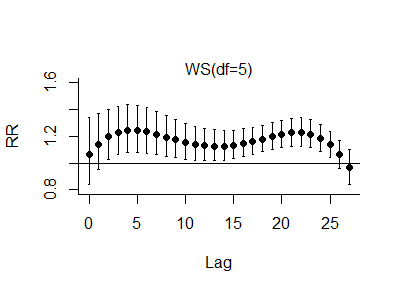

Supplement: Supplementary file 1 — Supplementary Material 1. [file 12889_2024_19030_MOESM1_ESM.docx]
